# Peripheral T-cell lymphoma, not otherwise specified- Case report

**DOI:** 10.1016/j.amsu.2020.05.035

**Published:** 2020-05-30

**Authors:** Nariman Khan, Collin Clay, Andrew Donati

**Affiliations:** Department of Internal Medicine, University of Texas Health Science Center, San Antonio, TX, USA

**Keywords:** T-cell lymphoma, Peripheral T-cell lymphoma, Lymphoepithelioid lymphoma, Malignancy, Angioimmunoblastic T-cell lymphoma

## Abstract

T-cell lymphomas are rare malignancies that are often found in older individuals. Here we present the case of a 63 year old female that presented with diffuse cervical, inguinal, and axillary lymphadenopathy with associated weight loss and night sweats. The patient underwent excisional lymph node biopsy which revealed a T-cell lymphoma with findings characteristic of peripheral T-cell lymphoma, not otherwise specified.

## Highlights

• EBV may be found positive in PTCL-NOS, Lennert lymphoma variant.
• There is frequent loss or decreased expression of CD5 and CD7 in PTCL-NOS.
• Excisional biopsy is necessary to diagnose malignancy if there is high suspicion and FNA/core biopsy are negative.

## 1 Introduction

T-cell lymphomas comprise less than 15 percent of non-hodgkin's lymphomas in the United States. There are two main types of T-cell lymphomas including peripheral T-cell lymphomas and T-lymphoblastic lymphoma/leukemia. Peripheral T-cell lymphomas include cutaneous T-cell lymphomas, adult T-cell leukemia/lymphoma, angioimmunoblastic T-cell lymphoma, enteropathy associated intestinal T-cell lymphoma, anaplastic large cell lymphoma, and peripheral T-cell lymphoma, not otherwise specified [1]. The prognosis of the lymphoma depends on its type, stage at diagnosis, and response to conventional therapy.

## 2 Case presentation

A 63 year old white female with past medical history significant for coronary artery disease with stent placement presented to the hospital with diffuse cervical, inguinal, and axillary lymphadenopathy that had been present for almost two years. The patient reported that her lymph nodes were progressively increasing in size and causing her to have dysphagia to solids and shortness of breath when lying flat. She also endorsed looser fitting clothes indicating weight loss and drenching night sweats.

The patient said that she first developed lymphadenopathy on the right side of her neck two years ago while working in a chicken farm with exposure to ammonia. She saw a physician for lymphadenopathy at that time but was told that her swollen lymph nodes were likely reactive from ammonia. The patient quit her job but the lymphadenopathy persisted.

Extensive work-up for the patient's lymphadenopathy was done with the highest suspicion for malignancy versus infectious etiology as the cause of the patient's presentation. Computed tomography of the chest showed diffuse cervical, mediastinal, and axillary lymphadenopathy with areas of necrosis and scattered groundglass opacities in the upper and lower lobes with multiple bilateral pulmonary nodules. Computed tomography of the abdomen and pelvis showed extensive abdominal, retroperitoneal, and pelvic lymphadenopathy, splenomegaly, and scattered sclerotic foci throughout the vertebral bodies and pelvis.

Infectious work-up including HIV, syphilis, histoplasma, tuberculosis, toxoplasma, blastomyces, brucella, bartonella, coccidioides, and HTLV I and II proved to be negative. However EBV viral capsid antibody, early antigen IgG, and nuclear antigen antibody were all found to be elevated.

Next, a fine needle aspiration and core biopsy were performed on the patient's left axillary node. Surprisingly the results proved to be negative for malignancy and the patient was diagnosed with granulomatous lymphadenitis with eosinophilic infiltrate. However, due to the patient's extensive lymphadenopathy an excisional lymph node biopsy of the left inguinal area was performed. The biopsy results showed T-cell lymphoma with Ebstein-Barr virus highlighting rare small-sized cells on immunoperoxidase studies. Polymerase chain reaction of the sample showed T-cell receptor beta and gamma gene rearrangement.

## 3 Discussion

The patient's excisional biopsy results showed T-cell lymphoma with the most likely differentials being peripheral T-cell lymphoma, not otherwise specified (PTCL-NOS) versus angioimmunoblastic T-cell lymphoma (AITL). The pathologist's report described the excised lymph node as completely effaced with increased vascularity and a mixed inflammatory infiltrate with numerous small collections of epithelioid histiocytes distributed throughout the lymph node. AITL, a subtype of mature peripheral T-cell lymphoma was considered due to the increased vascular proliferation and EBV (Ebstein-Barr virus) positive cells in loose clusters in the sample. However, its probability was lowered due to lack of aberrant expression of markers CD10, BCL6, CXCL13, and PD-1 by the neoplastic T cells which have a high sensitivity and specificity for AITL when combinantly detected [2]. The patient also did not present with any autoimmune findings such as autoimmune hemolytic anemia and immune thrombocytopenia or with polyclonal hypergammaglobulinemia, findings that are common in AITL patients [3].

PTCL-NOS are a group of heterogeneous diseases that involve the lymph nodes and extranodal sites that are diagnosed based on histopathology, aberrant T cell phenotype, and T-cell receptor rearrangement [4]. Variants of PTCL-NOS include lymphoepithelioid type, T-zone, and follicular. There is frequent loss or decreased expression of CD5 and CD7 and the most frequent phenotype is CD4+/CD8-but CD4+/CD8+ and CD4-/CD8-may also be seen [5]. Our patient expressed the CD4+/CD8+ T-cell phenotype with aberrantly dim expression of CD2, CD5, and CD7 on flow cytometry. Lymphoepithelioid or Lennert lymphoma, a subtype of PTCL-NOS was favored in this patient due to the presence of numerous epithelioid histiocytes in clusters throughout the lymph node. T-zone variant of PTCL-NOS was less likely due to lack of preservation of germinal centers in the lymph node [6].

Furthermore, this patient endorsed that her lymphadenopathy first started while she was working in a chicken farm with exposure to ammonia. It is known that ammonia is a corrosive substance that irritates body areas that it comes in contact with such as the lungs, skin, and eyes. However, ammonia is not known to be a carcinogen and likely was not the cause of this patient's malignancy [7]. She did not possess any risk factors for malignancy other than age.

## 4 Conclusion

The treatment of T-cell lymphoma depends on the type and includes chemotherapy, immunotherapy, targeted therapy, radiation, steroids, or stem cell transplantation. CHOP (cyclophosphamide, doxorubicin, vincristine, and prednisone) or EPOCH (etoposide, vincristine, doxorubicin, cyclophosphamide, and prednisone) chemotherapies are usually the initial regimens given to patients. Patients with relapsed disease are treated with ICE (ifosfamide, carboplatin, and etoposide) therapy [8]. The median life expectancy with PTCL-NOS is 2–3 years [9] and with AITL is 1–3 years [10]. At this time, the treatment for this patient's malignancy remains undecided due to funding issues but it is expected that she will receive CHOP therapy in the future.

## Provenance and peer review

Not commissioned, externally peer reviewed.
